# Negative Attitudes toward Older Workers and Hiring Decisions: Testing the Moderating Role of Decision Makers’ Core Self-Evaluations

**DOI:** 10.3389/fpsyg.2016.02057

**Published:** 2017-01-12

**Authors:** Ulrike Fasbender, Mo Wang

**Affiliations:** ^1^Centre for Diversity Policy Research and Practice, Oxford Brookes UniversityOxford, UK; ^2^Department of Management, Warrington College of Business Administration, University of Florida, GainesvilleFL, USA

**Keywords:** core self-evaluations, avoidance tendencies, hiring decisions, negative attitudes toward older workers, selection likelihood, hiring bias

## Abstract

Organizational hiring practices have been charged for unfair treatment on the grounds of age. Drawing on theories of planned behavior and core self-evaluations, this research investigated the impact of negative attitudes toward older workers on hiring decisions and examined the moderating role of decision-makers’ core self-evaluations. We tested our hypotheses based on a structured online questionnaire and a vignette study using a sample of 102 participants working in human resource management across different industries. As predicted, negative attitudes toward older workers were positively related to avoidance of hiring older people, which in turn was negatively related to the likelihood to select the oldest candidate. Because hiring decisions are not only about the hiring subject but also about the decision-maker, we tested the moderating role of decision-makers’ core self-evaluations. Results showed that core self-evaluations buffered the relationship between negative attitudes toward older workers and avoidance of hiring older people. Theoretical implications of the findings with regard to hiring decisions about older people and practical recommendations to improve diversity management strategies and age-balanced hiring practices in organizations are discussed.

## Introduction

Although age discrimination is against the law in many industrialized countries (Lahey, 2010), it is still a common phenomenon in hiring. A recent meta-analysis on age and reemployment success after job loss by Wanberg et al. (2016) revealed that older people receive fewer offers (ρ = -0.11), are less likely to obtain reemployment after job loss (ρ = -0.15), and take longer to find reemployment (reemployment speed: ρ = -0.17). In particular, people over the age of 50 suffer from longer unemployment periods. Organizational hiring practices have often been challenged for unfair treatment on the grounds of age (Truxillo et al., 2015). Although many older people wish to work until an older age (Wöhrmann et al., 2016), they may be denied the opportunity due to relevant decision-makers’ negative attitudes toward older workers. There is a substantial amount of research showing that older people face disadvantages in applying for jobs. In fact, existing field experiments based on correspondence testing almost always showed substantial age discrimination in hiring processes (e.g., Gringart and Helmes, 2001; Riach and Rich, 2010; Albert et al., 2011; Neumark et al., 2016).

Drawing on the theory of planned behavior (Ajzen, 1991), one explanation for the unfair treatment of older applicants is that negative attitudes toward older workers reign within organizations and lead to discrimination against them (Shore and Goldberg, 2005). Thus, age-related negative attitudes are thought to underlie discriminatory behaviors such as lower hiring rates for equally qualified older (vs. younger) job applicants. Despite their centrality for explaining age discrimination, they have not yet received much attention in organizational research on age bias in hiring (Shore and Goldberg, 2005; Finkelstein and Farrell, 2007; Finkelstein et al., 2015). There are numerous studies that investigated the existence of negative attitudes toward older workers (for review see for example Posthuma and Campion, 2009) but only few studies show their direct link to hiring decisions and the underlying mechanisms. For example, a field study by Lu et al. (2011) showed that positive attitudes toward older workers were positively related to mangers’ intentions to hire older people. Further, experimental research by Krings et al. (2011) revealed that participants, who had more favorable attitudes toward older workers (i.e., high competence) were more likely to suggest an older applicant for a job interview. However, these studies neglect the role that decision-makers’ self-concept plays during the hiring process.

Taking this into account, we argue that decision-makers’ core self-evaluations – defined as a fundamental, bottom-line assessment of one’s ability, merit and efficacy (Judge et al., 2003) – is a positive self-concept and can buffer the impact of negative attitudes toward older workers on decision-makers’ avoidance tendencies, which in turn can determine their actual hiring decisions. Although the idea that decision-makers’ self-concept and social identity can jointly influence hiring decisions has been highlighted before (Lewis and Sherman, 2003), no study has examined how core self-evaluations may interfere the relationship between negative attitudes toward older workers and avoidance of hiring older people.

To address this research gap, we investigate negative attitudes toward older workers in hiring decisions and examine the moderating role of core self-evaluations. In particular, we make two contributions to the literature. First, we explore the process of age discrimination in hiring by disentangling discriminatory attitudes, intentions, and behavior. In particular, we reveal avoidance tendencies as a mediator between negative attitudes toward older workers and hiring decisions. Second, we test the moderating role of decision-makers’ core self-evaluations in buffering the negative impact of age-related negative attitudes in the workplace. As most research demonstrates the problem of discriminatory behavior rather than to facilitate its prevention, identifying potential moderators is essential in challenging age discrimination in hiring. As a result, our study has important implications to organizational practice in terms of training and development of decision-makers with regard to their self-concept.

### Theoretical Background

#### Hiring Decisions about Older People

Because chances of being hired are lower for older people, researchers have been searching for potential factors and circumstances to counter disadvantages that older people have to face in hiring. Previous research has mainly investigated environmental circumstances influencing employers’ hiring decisions about older people (Earl et al., 2015). As such, scholars have indicated that experienced difficulties in recruiting or labor shortages in general are likely to facilitate hiring older people (Taylor et al., 2013). On the other hand, high cost pressure is likely to lower older people’s hiring chances as that has been found to lower the probabilities for older workers to receive training and development (e.g., Erber and Danker, 1995; Loretto and White, 2006). Also, legal approaches (i.e., stronger age discrimination laws at the state level) aiming at improving equal opportunities for younger and older people have been found to be rather dysfunctional for older people in times of low labor demands (i.e., economic recession) (Neumark and Button, 2014). Therefore, research needs to continue its search for relevant factors underlying hiring decisions about older people. In the current study, we investigate negative attitudes toward older workers in hiring decisions and highlight the moderating role of core self-evaluations. Based on the theory of planned behavior (Ajzen, 1991) and the core self-approach (Judge et al., 1997), we argue that hiring decisions are not only about finding the most suitable candidate for a certain job vacancy, but also about one’s internal evaluation of the potential consequences of the hiring decision for one’s self-concept.

#### Negative Attitudes toward Older Workers and Hiring Decisions

According to the theory of planned behavior (Ajzen, 1991), behavior (i.e., the hiring decision) is determined by intention (i.e., the hiring intention) as the most proximal predictor, which in turn is entirely influenced by attitudes, norms and perceived behavioral control toward the behavior. Ajzen’s theory of planned behavior is a well-established conceptual framework that has been frequently used to explain hiring decisions about members of discriminated groups (e.g., Lu et al., 2011; Ang et al., 2015; Araten-Bergman, 2016). In particular, attitudes (i.e., negative attitudes toward older workers) have been highlighted as important mechanism to influence the decision-making process of hiring older people (e.g., Posthuma and Campion, 2009; Truxillo et al., 2015).

In the hiring context, attitude reflects decision-makers’ affective or cognitive evaluation of the hiring targets (e.g., older people). For example, one decision-maker may think older workers are harder to train for jobs; whereas another might believe older people are more dependable at work (Fasbender, 2016). Further, it is assumed that one’s positive attitudes lead to approach, whereas one’s negative attitudes lead to avoid certain behaviors, such as hiring older people. This notion is partly supported by Lu et al. (2011), who found that managers’ positive attitudes toward older workers were positively related to their intention to hire older people as opposed to avoid hiring them. Avoidance of hiring older people can be conceptualized as the intention not to hire older people, which eventually leads to an actual decision of not hiring a particular older person. Meta-analytical findings reveal that negative attitudes are more powerful in predicting important behavioral outcomes then positive attitudes do (Meisner, 2012). We therefore propose that negative attitudes toward older workers are likely to increase decision-makers’ avoidance tendencies of hiring older people. In turn, it is likely that avoidance tendencies result in actual behavior, such as selecting younger candidates instead of an equally qualified older candidate in the hiring situation. To sum up, our first two hypotheses read:

H1: Negative attitudes toward older workers are positively related to avoidance of hiring older people.
H2: Avoidance of hiring older people is negatively related to the likelihood of selecting the equally qualified oldest candidate in the hiring situation.

Having introduced avoidance tendencies as the underlying mechanism, we draw a link between negative attitudes toward older workers and selecting the oldest candidate in the hiring situation. Taking Hypotheses 1 and 2 together, we assume that there is a negative relationship between negative attitudes toward older workers and selection likelihood, which is expected to be mediated by avoidance of hiring older people. Previous research partly supports this notion. Early research by Perry et al. (1996) found that bias against older workers was related to lower evaluation of an older applicant among business students. Similarly, Krings et al. (2011) showed that biased beliefs about older workers led to age discrimination at selection among business students and also among HR professionals. These studies point at a negative relationship between negative attitudes toward older workers and selection likelihood. However, based on the theory of planned behavior (Ajzen, 1991), we argue that the hiring decision is not directly undertaken but intended prior to the actual decision. Thus, we propose an indirect effect of negative attitudes toward older workers and selection likelihood via avoidance of hiring older people.

H3: There is a negative relationship between negative attitudes toward older workers and the likelihood of selecting the equally qualified oldest candidate in the hiring situation, which is mediated by avoidance of hiring older people.

#### Core Self-Evaluations as Moderator

Core self-evaluations can be defined as a positive self-concept referring to basic conclusions that individuals hold about themselves (Judge and Bono, 2001). Initially, Judge et al. (1997) have introduced core self-evaluations as a superordinate construct capturing self-esteem, generalized self-efficacy, locus of control, and emotional stability traits. Based on Cattell’s (1965) personality theory, these traits were identified following three important criteria: evaluation-focus, fundamentality, and scope. Of the four traits, self-esteem has been described as “the most fundamental manifestation of core self-evaluations as it represents the overall value that one places on oneself as a person” (Judge and Bono, 2001, p. 80). Further, generalized self-efficacy constitutes one’s ability to perform, cope, and be successful; internal locus of control reflects the belief of being able to control a broad array of factors in one’s live; and finally, high emotional stability (vs. low neuroticism) refers to being confident, secure and steady (Judge and Bono, 2001). Since its initial introduction, there has been a substantial amount of evidence supporting the construct validity of core self-evaluations (e.g., Judge et al., 2003; Gardner and Pierce, 2009; Stumpp et al., 2010). Besides, core self-evaluations have been found to be powerful in predicting a range of important work-related outcomes. Results of a meta-analysis by Chang et al. (2012) suggest that core self-evaluations hold positive relationships with job satisfaction (ρ = 0.36), goal commitment (ρ = 0.42), intrinsic motivation (ρ = 0.33), task performance (ρ = 0.19) and organizational citizenship behaviors (ρ = 0.23) but negative relationships with turnover intentions (ρ = -0.26) and counterproductive work behavior (ρ = -0.17).

Despite its importance for individuals’ work-related outcomes, research has so far neglected the impact of core-self evaluations on others in the workplace. Particularly, it is unknown to what extent decision-makers’ core self-evaluations are related to hiring older people. Addressing the decision-maker perspective, we argue that as people often derive some aspects of their self-concept from the groups they belong to (Tajfel and Turner, 1986), they are motivated to achieve self-enhancement and self-esteem by developing a positive distinctiveness between the ingroup and outgroup (i.e., the self-esteem hypothesis; Abrams and Hogg, 2010). As such, decision-making is influenced by people’s motivation to maximize their positive self-concept. In order to maintain a positive self-concept, people are keen on seeing their ingroup members in the most favorable light possible, whereas outgroup members are perceived as a potential threat to one’s self. Core self-evaluations can function as a source of self-protection against external threats (Judge et al., 1997). Therefore, the perceived threat from outgroup members in a hiring decision is likely to be higher for people with low core self-evaluations. In other words, people with low core self-evaluations are particularly vulnerable to discriminate against outgroup members in making hiring decisions when holding negative attitudes toward them, whereas people with high core self-evaluations are less vulnerable.

Previous research has mainly addressed the impact of self-esteem on discriminatory behavior. A meta-analysis revealed that both low and high self-esteem individuals tend to hold ingroup bias (Aberson et al., 2000). There is also research showing that emotional stability may impact managers’ hiring decisions about members of discriminated groups (i.e., comparing native and immigrant job candidates) (Horverak et al., 2013). Core self-evaluations as a superordinate construct consisting of self-esteem, self-efficacy, locus of control, and emotional stability can be regarded as the baseline for any self-categorization process. We therefore argue that decision-makers’ core self-evaluations can buffer the impact of negative attitudes toward older workers on decision-makers’ avoidance tendencies, which in turn determine their actual hiring decisions. To sum up, our fourth hypothesis reads:

H4: Core self-evaluations moderate the relationship between negative attitudes toward older workers and avoidance of hiring older people in a way that the positive relationship is weaker when core self-evaluations are high (vs. low).

## Materials and Methods

### Design, Sample, and Procedure

We used a structured online questionnaire and a vignette study to collect the data. A vignette study was designed to understand to what extent the hiring decision may be influenced by applicants’ age as a proxy for discriminatory behavior. Vignette methodology has been described as a systematic approach in achieving both internal and external validity (Aguinis and Bradley, 2014). In the current study, participants were given a job description for a managerial position vacancy for a fictitious company and were required to choose the best candidate based on their Curriculum Vitae (CV) for this vacancy. Three candidates’ CVs were designed to indicate equivalent work experience in a series of pilot studies (see Materials and Piloting below), but the candidates’ ages were varied (i.e., 38, 49, and 60 years). Participants were then asked to prioritize CVs for hiring. Whether the participant selected the 60-year old candidate for the vacancy constituted the dependent variable in this study.

Potential participants living in the United States were invited to take part using a professional research platform (i.e., Call For Participants), where they were offered gift voucher as compensation for their participation. As with other online crowdsourcing mechanisms (i.e., Amazon’s Mechanical Turk), the data obtained can be regarded as reliable as the data collected via traditional methods (Buhrmester et al., 2011). Participants were included if they currently worked in human resource management and had hiring power on their jobs. In other words, our sample contains human resource managers dealing with hiring decisions on a daily basis. Also, we checked the amount of time that participants spent on the task and eliminated participants who spent very little time to complete the study in order to ensure sufficient data quality. In total, 165 participants completed the study across different industries ranging from industrial goods to technology, media and telecommunications. Of these, 51 participants were excluded because they reported to have no hiring power at work. Another 12 participants were excluded because they spent very little time to complete the study. Overall, the final sample consisted of 102 participants of which 64 (62.7%) were male and 87 (85.3%) held a higher education degree. Participants’ ages ranged from 22 to 52 years, with a mean age of 36.10 years (*SD* = 6.64). Of the excluded participants, 35 (55.56%) were male and 48 (76.19%) held a higher education degree; the percentage of participants did not statistically differ from the percentage of participants in the final sample (sex: χ^2^ [1, *N* = 165] = 0.84, *p* = 0.36; education: χ^2^[1, *N* = 165] = 2.17, *p* = 0.14). Further, they were a bit younger compared to participants of the final sample [*t*(163) = 2.93, *p* < 0.01]; their ages ranged from 25 to 46 with a mean age of 33.19 years (*SD* = 5.37).

### Materials and Piloting

Following the procedure of Derous et al. (2012), two pilot studies were conducted to ensure the equivalence of the templates used as study material. Three templates were designed so that work experience over the last 10 years shown on the CV corresponds to the job description used in the main study. The work experience was matched to the job description based on information available on common job search platforms (e.g., Indeed and Monster). *A priori* power analysis (calculated in G^∗^Power) revealed a sample size of at least 42 participants to detect a small to medium effect (*f* = 0.20) for the one-way repeated measures Analysis of Variance (ANOVA) with a test power of 80%. Participants of the pilot samples were recruited via the same professional research platform (i.e., Call For Participants) used in the main study. The initial pilot sample of 50 participants evaluated the different templates presented according to the job description. Participants have been asked to evaluate the person-job fit with five items on a 7-point Likert-type scale (1 = *not at all* to 7 = *extremely*). One example item was: “Candidate [X] is qualified for this job.” One-way repeated measures analysis of variance (ANOVA) revealed significant differences between the three templates [*F*(1.71,83.83) = 6.54, *p* < 0.01]. *Post hoc* tests were conducted to compare all pairs of templates. Results of pairwise *t*-tests showed that the templates were slightly different from each other as there were significant differences between one template with the other two [Template 2: *M* = 6.01, *SD* = 0.70 and Template 1: *M* = 6.24, *SD* = 0.64, *t*(49) = 2.01, *p* < 0.05; Templates 2 and 3: *M* = 6.38, *SD* = 0.70, *t*(49) = -3.31, *p* < 0.01]. Therefore, the templates were revised and tested for a second time. To revise the templates, the work experience presented was again carefully matched to the job description. Further, more attention was paid to small differences in language use. The second pilot sample consisted of 42 participants. This time, results of the one-way repeated measures ANOVA did not show significant differences between the templates [Template 1: *M* = 6.03, *SD* = 0.72; Template 2: *M* = 6.02, *SD* = 0.83; Template 3: *M* = 6.03, *SD* = 0.87, *F*(2,82) = 0.02, *p* = 0.98]; these templates were used in the main study.

### Measures

#### Negative Attitudes toward Older Workers

Participants’ negative attitudes toward older workers were measured by the means of their responses to the negatively framed items of the Beliefs about Older Workers Questionnaire from Hassell and Perrewe (1995). Respondents rated the degree to which they were holding negative beliefs about older people in the work context on a seven-point scale ranging from 1 (*strongly disagree*) to 7 (*strongly agree*). One example item was: “Most older workers cannot keep up with the speed of modern industry.” The 15 items yielded a good internal consistency (Cronbach’s α = 0.88) in this study.

#### Core Self-Evaluations

Core self-evaluations were measured by the means of the Core Self-Evaluations Scale (Judge et al., 2003). Respondents rated their endorsement to several statements about themselves on a seven-point scale ranging from 1 (*strongly disagree*) to 7 (*strongly agree*). One example item was: “I am confident I get the success I deserve in life.” The scale yielded an acceptable internal consistency (Cronbach’s α = 0.71) in this study.

#### Avoidance of Hiring Older People

Avoidance of hiring older people was measured by the means of three items adapted from Hutchison et al. (2010). Respondents rated the degree they were avoiding to hire older people if they can on a 5-point scale ranging from 1 (*strongly disagree*) to 5 (*strongly agree*). The three items were: “If I had a choice I would rather not hire an older person.”, “If I can avoid hiring older people, I do.”, and “I would want to avoid hiring an older person.” The scale yielded a good internal consistency (Cronbach’s α = 0.81) in this study.

#### Selection of the Oldest Candidate

Selection of the oldest candidate was measured with one item related to the task of prioritizing applicants’ CVs for hiring. The responses to this task were coded as a dichotomous variable (i.e., 0 = *not selecting the oldest candidate*; 1 = *selecting the oldest candidate*). Because the different CVs were tested for equivalence with regard to applicants’ work experience (see Materials and Piloting above), not selecting the oldest candidate serves as a proxy for discriminatory behavior. Of the 102 participants, 25 participants (24.5%) ranked the oldest candidate to be the most suitable person for the described job vacancy, which is below the chance level (33.3%) if no discrimination was present.

#### Control Variables

As the outcome variable may be affected by participants’ age, sex, and education, we included these variables as covariates in the analyses. In addition, we controlled for social desirability using a 13-item scale (Reynolds, 1982) to provide a more conservative examination of the hypothesized relationships and to gauge the extent to which our results might be biased by common method bias (Wang et al., 2015).

## Results

### Preliminary Analysis

The descriptive statistics and correlations of all study variables are shown in **Table 1**. Avoidance of hiring older people was positively correlated with negative attitudes toward older workers (*r* = 0.62, *p* < 0.01) providing initial support for Hypothesis 1. Further, avoidance of hiring older people was negatively related to participants’ age (*r* = -0.25, *p* < 0.05) and to core self-evaluations (*r* = -0.27, *p* < 0.01). Correlations with sex, education, and social desirability were weaker and not significant. Selection of the oldest candidate was negatively related to avoidance of hiring older people (*r* = -0.16, *p* < 0.10), providing initial support for Hypothesis 2. Given the low base rate of selecting the oldest candidate, this relationship can be regarded as quite substantial. Correlations with selection of the oldest candidate were weaker (and not statistically significant) for age, sex, education, social desirability, core self-evaluations, and negative attitudes toward older workers.

**Table 1:** Means, standard deviations, and correlations for all of the variables (*N* = 100–102).

Variable	*M*	*SD*	1	2	3	4	5	6	7	8
(1) Age	36.10	6.64	-							
(2) Sex (1 = male)	0.63	0.49	0.10	-						
(3) Education (1 = university degree)	0.85	0.36	-0.15	-0.03	-					
(4) Social desirability sum score	6.95	2.07	0.22^∗^	0.10	0.00	-				
(5) Core self-evaluations	4.43	0.78	0.23^∗^	0.02	0.27^∗∗^	0.37^∗∗^	-			
(6) Negative attitudes toward older workers	3.42	0.67	-0.10	0.10	0.15^†^	-0.17^†^	-0.10	-		
(7) Avoidance of hiring older people	3.15	1.00	-0.25^∗^	0.10	0.06	-0.14	-0.27^∗∗^	0.62^∗∗^	-	
(8) Selection of the oldest candidate (1 = yes)	0.25	0.43	-0.00	0.06	-0.02	-0.07	-0.01	-0.05	-0.16^†^	-

*^†^*p* < 0.10, ^∗^*p* < 0.05, ^∗∗^*p* < 0.01.*

### Hypothesis Testing

**FIGURE 1:**
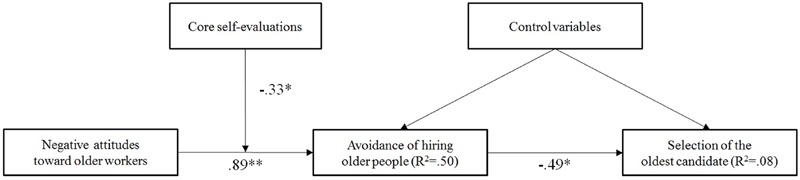
**Estimated path model showing relationships between negative attitudes toward older workers, core self-evaluations, avoidance of hiring older people, and selection of the oldest candidate with unstandardized coefficients (*N* = 100).** ^∗^*p* < 0.05, ^∗∗^*p* < 0.01.

To investigate the hypothesized relationships between negative attitudes toward older workers, core self-evaluations, avoidance of hiring older people, and selecting the oldest candidate, we used path analysis to analyze the data. In Mplus 7.31 (Muthén and Muthén, 2015), we applied robust maximum likelihood estimator (MLR) and logistic link function because the dependent variable (i.e., selection of the oldest candidate) was dichotomous in nature (Yuan and Bentler, 2008). We tested our hypotheses by including all variables and hypothesized effects simultaneously in the model. We then compared the partial against the full mediation model (the partial mediation model has a direct effect from negative attitudes toward older workers to the dependent variable). The fit indices for the two models are presented in **Table 2**. Due to the use of MLR estimator, chi-square based fit indices are not available to evaluate model fit. Therefore, we rely on information criteria [i.e., Akaike Information Criterion (AIC), Bayesian Information Criterion (BIC), and Sample-size Adjusted Bayesian Information Criterion (SABIC)] to determine which model fit to the data better while being parsimonious (Preacher et al., 2007). Lower values of AIC, BIC, and SABIC indicate the most optimal balance between model fit and parsimony. The results indicated that the hypothesized full mediation model had lower values of AIC, BIC, and SABIC as compared to the partial mediation model and can therefore be considered as the most parsimonious and better-fitting model. The estimated path model showing relationships between control variables, negative attitudes toward older workers, core self-evaluations, avoidance of hiring older people, and selecting the oldest candidate is presented in **Figure 1**. With regard to the control variables, only age was negatively related to avoidance of hiring older people (γ = -0.03, *p* < 0.01), indicating that older decision-makers reported lower avoidance tendencies toward hiring older people.

**Table 2:** Fit indices for structural models (*N* = 100).

Model	Log likelihood	AIC	BIC	SABIC
Mediation model (partial) with interaction	**-160.809**	353.617	395.300	344.768
Mediation model (full) with interaction	**-**161.001	**352.003**	**391.080**	**343.706**

*Bold entries signify the lowest values in each column, AIC, Akaike Information Criteria; BIC, Bayesian Information Criteria; SABIC, Sample-size Adjusted Bayesian Information Criteria.*

Hypotheses 1–3 addressed the relationships between negative attitudes toward older workers and selection of the oldest candidate. The path coefficients suggested that negative attitudes toward older workers were positively related to avoidance of hiring older people (γ = 0.89, *p* < 0.01), supporting Hypothesis 1. This indicates that decision-makers, who reported more negative attitudes toward older workers, were more likely to avoid hiring them. In turn, avoidance of hiring older people was negatively related to selecting the oldest candidate (γ = -0.49, *p* < 0.05, OR = 0.61). This result supports Hypothesis 2 and indicates that decision-makers, who reported one-unit higher avoidance tendencies toward hiring older people, were 0.61 times less likely to select the oldest candidate in this study. With regard to the relationship between negative attitudes toward older workers and selecting the oldest candidate, the compound coefficient suggested that there was a negative indirect effect via avoidance of hiring older people (*indirect effect* = -0.44, *p* < 0.05). This result supports Hypothesis 3 and indicates that there was a negative relationship between negative attitudes toward older workers and selecting the oldest candidate, which is mediated by decision-makers’ avoidance of hiring older people.

**FIGURE 2:**
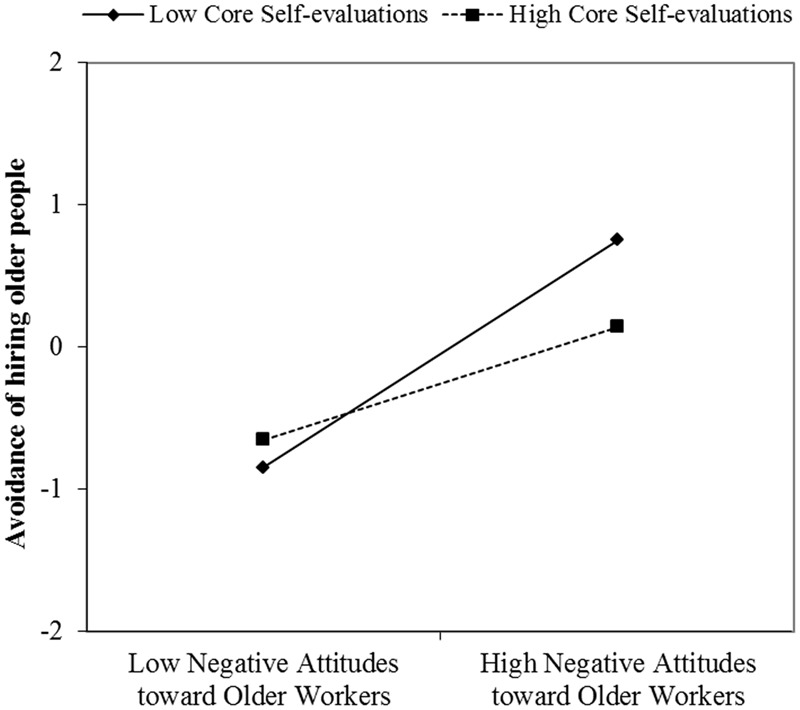
**Core self-evaluations moderate the relationship between negative attitudes toward older workers and avoidance of hiring older people**.

Hypotheses 4 addressed the moderating role of core self-evaluations. The estimated coefficients showed that core self-evaluations moderated the relationship between negative attitudes toward older workers and avoidance of hiring them (γ = -0.33, *p* < 0.05). Simple slope analysis revealed that the positive relationship was weaker when core self-evaluations were high (*simple slope* = 0.63, *z* = 3.93, *p* < 0.01) than when core self-evaluations were low (*simple slope* = 1.16, *z* = 7.20, *p* < 0.01). As can be seen in **Figure 2**, high (vs. low) core self-evaluations buffered the positive relationship between negative attitudes toward older workers and avoidance of hiring older people, supporting Hypothesis 4.

## Discussion

The aim of the current study was to investigate the impact of negative attitudes toward older workers on hiring decisions and to examine the moderating role of decision-makers’ core self-evaluations. We tested our hypotheses based on a structured online questionnaire and a vignette study using a sample of 102 decision-makers with hiring power across different industries. Results of the vignette study revealed that only 24.5% of participants ranked the oldest candidate to be the most suitable person for a given job vacancy, which is below the chance level of 33.3%. The selection likelihood can be regarded as a proxy for discriminatory behavior because the three candidates were equally qualified.

We found that negative attitudes toward older workers were positively related to avoidance of hiring older people, which in turn was negatively related to the likelihood to select the oldest candidate. Further, the present study revealed an indirect effect of negative attitudes toward older workers and selection likelihood via avoidance of hiring older people. Consistent with the literature on the theory of planned behavior (Ajzen, 1991) and hiring decisions about members of discriminated groups (e.g., Perry et al., 1996; Krings et al., 2011; Lu et al., 2011; Ang et al., 2015; Araten-Bergman, 2016), these findings confirm that negative attitudes toward older workers as hiring subjects lead to avoidance tendencies, which in turn result in an actual decision not to hire older people.

Moreover, the current study revealed the moderating role of decision-makers’ core self-evaluations in hiring decisions about older people. As predicted, we found that core self-evaluations buffered the relationship between negative attitudes toward older workers and avoidance of hiring older people. In line with the literature on self-concept, social identity, and hiring decisions (Lewis and Sherman, 2003), this finding highlights decision-makers’ core self-evaluations as a relevant mechanism in challenging age discrimination in hiring. As older people can be a threat to younger and middle-aged decision-makers’ self-concept, people with low core self-evaluations are particularly susceptible to discriminate against others when holding negative attitudes toward them, whereas people with high self-evaluations are less susceptible to discriminate against others during hiring decisions. Thus, decision-makers’ high (vs. low) core self-evaluations can reduce the impact of negative attitudes toward older workers on decision-makers’ avoidance tendencies, which in turn determine their actual hiring decisions.

### Theoretical and Practical Implications

The findings of the current study extend previous research on hiring decisions about older people and offer relevant theoretical and practical implications. With regard to theory, we extend previous knowledge on age discrimination in hiring by disentangling discriminatory attitudes, intentions, and actual decision-making. Although negative attitudes toward older workers are thought to underlie discriminatory behaviors such as lower hiring rates for equally qualified older (vs. young and middle-aged) job applicants, they have not received much attention in organizational research on hiring bias (Shore and Goldberg, 2005; Finkelstein and Farrell, 2007). Numerous studies have investigated the existence of negative attitudes toward older workers (Posthuma and Campion, 2009) but very few studies show the direct link to hiring decisions and its underlying mechanisms. The present study confirms the previous notion that hiring decisions about older people are intended before the actual decision is made and that this hiring intention (i.e., avoidance vs. approach tendency) is strongly influenced by decision-makers’ negative attitudes toward older workers as hiring subject.

Moreover, the present study highlights that hiring decisions are not only about finding the most suitable candidate for a certain job vacancy but also about protecting one’s self-concept. Most notably, our findings shed light on the moderating role of decision-makers’ core self-evaluations. In particular, we reveal that decision-makers’ core self-evaluations can buffer the impact of negative attitudes toward older workers on decision-makers’ avoidance tendencies, which in turn determine their actual hiring decisions. This supports the previous notion that decision-making is influenced by people’s motivation to maximize their positive self-concept (Lewis and Sherman, 2003). In general, people are keen on seeing their ingroup members in the most favorable light possible, whereas outgroup members are perceived as a potential threat to one’s self. This potential threat is stronger for people with low (vs. high) core self-evaluations. In hiring, decision-makers with low core self-evaluations are therefore more susceptible to discriminate against older people when holding negative attitudes toward them as compared to decision-makers with high core self-evaluations. Future studies need to replicate our study findings in order to consolidate theoretical implications about the moderating role of decision-makers’ core self-evaluations.

With regard to practice, the current study contributes to the improvement of diversity management strategies in organizations facilitating age-balanced hiring practices. To begin with, organizations need to tackle the issue of often existing negative attitudes toward older workers. As such, negative attitudes toward older workers should be reduced and positive attitudes toward older workers should be encouraged, for instance, by shaping a positive age climate and an age-friendly organizational culture leading to an appreciation of age diversity at work. Previous research has shown that intergenerational contact may be able to facilitate positive views toward older people at work (Iweins et al., 2013; Henry et al., 2015). In the workplace, regular and high quality exchange among decision-makers with different ages may be therefore effective in transforming negative attitudes into positive views toward older workers.

Another angle to look at age-balanced hiring practices, is to focus on decision-makers’ self-concept. Because hiring decisions are not only about the hiring subject but also about the decision-maker, it is important to ensure that decision-makers carry positive basic conclusions about themselves reducing the risk of being vulnerable for discriminatory mechanisms in hiring. This may be achieved by selecting or promoting decision-makers based on their core self-evaluations. However, Chang et al. (2012, p. 114) point out that “researchers and practitioners must ascertain whether the use of CSE (core self-evaluations) leads to adverse impact,” before using such measures for staffing. An alternative may be to train decision-makers’ core self-evaluations. Although core self-evaluations are defined as a superordinate construct capturing self-esteem, generalized self-efficacy, locus of control, and emotional stability traits (i.e., stable characteristics), it is almost certain that core self-evaluations are malleable and can change over time. For instance, research has reported changes in self-esteem (Orth et al., 2010), self-efficacy (e.g., West et al., 2008) and emotional stability traits (Roberts et al., 2006) over time. Thus, workplace interventions (e.g., personal training and development activities) may be able to enhance core self-evaluations among decision-makers and in turn support positive organizational outcomes. Further research is needed to assess the effectiveness of these interventions and to estimate to what extend core self-evaluations can be changed in the workplace.

### Limitations and Directions for Future Research

Notwithstanding the theoretical and practical implications of our findings, we address the limitations of this research and highlight directions for future research. First, the cross-sectional design does not allow for causal inferences. In fact, it is possible that the relationships between negative attitudes toward older workers, decision-makers’ core self-evaluations and hiring decisions about older people are bi-directional. However, the possible reverse causation cannot explain the interaction effect between negative attitudes toward older workers and decision-makers’ core self-evaluations on hiring decisions about older people. Nevertheless, future studies should adopt longitudinal and (quasi-)experimental research designs to allow for more conclusive findings.

Second, given that our study variables were assessed via self-reported data, common-method bias could be a concern. However, we controlled for social desirability in our analyses, which had a rather low correlation with avoidance of hiring older people as mediator (*r* = -0.17) and selecting the oldest candidate as outcome variable (*r* = -0.07). Also, in our final path model, social desirability has not been found to significantly predict the mediator or the outcome variable. This partly reduces the concern for common-method bias, as the covariation of social desirability and self-reported data represents a rather small systematic error variance explained by the common rating source (Smith and Ellingson, 2002; Wang et al., 2015).

Third, the number of vignettes has been limited to three candidates (i.e., aged 38, 49, and 60 years), which oversimplifies the hiring decision and therefore may reduce external validity. Future research should replicate our study findings using a larger number of vignettes in order to rule out the potential concern for external validity. Future studies can also vary vignettes by using additional person characteristics (e.g., gender, race/ethnicity, social status) to understand the intersectionality of social categorizations as previous research has revealed double jeopardy against applicants having a multiple stigmatized background (Derous et al., 2012). In addition, hiring decisions about older people may depend on the job type. For example, previous research has shown that age-related hiring bias may differ in relation to whether the job role is of low or high status (Abrams et al., 2016). Varying candidates’ profiles as well as investigating different types of jobs can help to understand the complex interplay between job candidate, job type, and decision-maker during hiring decisions.

Fourth, the present study has mainly focused on the fit between the candidate and the job. Even if a person is hired because of his or her qualifications with regard to the demands of the job, there is no guarantee that this person will fit to the organization. More recent approaches on hiring decision-making emphasize on the importance of person-organization fit, describing the compatibility between employees and the organization, which has been found to predict relevant work attitudes (ρ = 0.31; including job satisfaction and organizational commitment), job performance (ρ = 0.15; including task performance and contextual performance such as organizational citizenship behavior), and turnover (ρ = 0.24), respectively (Arthur et al., 2006). Given the importance of these outcome variables, future research should investigate the impact of negative attitudes on the decision-making about hiring older people with regard to the varying organizational contexts. Also, it would be worth investigating how shared negative attitudes in the organization (e.g., age discrimination climate; Kunze et al., 2011) influence older workers’ employment-related decisions (e.g., withdrawal and early retirement; Zaniboni, 2015; Griffin et al., 2016), as well as to what extent the decision-makers’ core self-evaluations can moderate these effects.

Moreover, the current study leaves some issues unaddressed and suggests directions for further investigation. As this study was tailored toward the cultural environment of the United States, future research needs to replicate our findings in other countries. Particularly, it is relevant to explore whether the link between negative attitudes toward older workers, core self-evaluations, and hiring decisions about older people are generalizable across different cultures. Recently, North and Fiske’s (2015) cross-cultural meta-analysis found relevant differences between Eastern and Western cultures regarding their attitudes toward older people, which may be reflected in hiring decisions. Also, the impact of core self-evaluations on hiring decisions is expected to differ across cultures. Some scholars have stated that people’s self-concept should be more influential in individualistic than in collectivistic cultures (e.g., Markus and Kitayama, 1998; Judge and Hurst, 2008), yet, Chang et al. (2012) meta-analytical findings suggest that the relationships between employees’ core self-evaluations and different work-related outcomes were stronger for collectivistic (vs. individualistic) cultures. Thus, future research ought to consider cultural differences in understanding the relationships between decision-makers’ core self-evaluations, their negative attitudes toward older workers, and decisions about hiring them. In addition, future research could explore different moderators as a means to inhibit age discrimination in hiring, such as decision-makers’ age group salience, their motivation to respond without prejudice, and organizational values and norms.

## Ethics Statement

This study was carried out in accordance with the recommendations of the University Research Ethics Committee of Oxford Brookes University with an informed consent from all study participants. Oxford Brookes’ University Research Ethics Committee has a multi-disciplinary membership that includes academic researchers (staff and students) from across the Faculties and non-research lay members from within and outside the University. It has specific responsibility for reviewing research that involves human participants, data or material, including both approving proposed research studies prior to the commencement of data collection and monitoring the progress of research that it has approved, to ensure compliance with approved ethics procedures. The University adheres to the principles of research ethics as laid out by the Economic and Social Research Council (ESRC 2015) – the UK’s largest research and training organization addressing economic and social concerns.

## Author Contributions

UF: Research design, data collection, data analysis, theorizing and writing. MW: Contribution to research design, contribution to data analysis, contribution to theorizing and writing.

## Conflict of Interest Statement

The authors declare that the research was conducted in the absence of any commercial or financial relationships that could be construed as a potential conflict of interest.
